# Intramedullary sealing with a bone plug in total knee arthroplasty to reduce blood loss: a meta-analysis of randomized controlled trials

**DOI:** 10.1186/s13018-019-1141-x

**Published:** 2019-04-08

**Authors:** Varah Yuenyongviwat, Pakjai Tuntarattanapong, Khanin Iamthanaporn, Theerawit Hongnaparak, Boonsin Tangtrakulwanich

**Affiliations:** Department of Orthopedics, Faculty of Medicine, Prince of Songkla University, Hat Yai, Songkhla 90110 Thailand

**Keywords:** Intramedullary sealing, Bone plug, Total knee arthroplasty

## Abstract

**Background:**

An intramedullary guide is an instrument that surgeons use to align the distal femoral cut. The opening may become a channel that drains intramedullary blood to the knee joint after surgery if left open during surgery. The authors aimed to evaluate the effects of an intramedullary bone plug with respect to postoperative blood loss from a meta-analysis.

**Methods:**

The authors performed a systematic review and meta-analysis to compare a sealed opening using an intramedullary bone plug with no bone plug. PubMed, Ovid, Embase, and Cochrane Library were used to identify all publications before May 2018. All of the included studies were evaluated for bias and heterogeneity.

**Results:**

Six hundred and thirty-six patients from four randomized controlled trials were included in this meta-analysis. The pooled results demonstrated that patients with intramedullary plug had lower rates of blood transfusion and lower level of reduced postoperative hemoglobin than patients in whom the intramedullary canal was not plugged.

**Discussion:**

This meta-analysis demonstrated the benefit of intramedullary sealing with a bone plug in total knee arthroplasty with respect to decreased postoperative blood loss.

## Background

Decreasing the blood loss and transfusion rate may decrease postoperative complications after total knee arthroplasty (TKA) [1]. Many methods have been mentioned to decrease blood loss during and after TKA, such as tranexamic acid [2], fibrin sealant [3], and positioning of knee flexion after surgery [4].

An intramedullary guide is an instrument used by surgeons to align the distal femoral cut. The drawback of this instrument is the necessity to penetrate the intramedullary canal from the distal femur that leaves an opening at the distal femur. This opening can be a channel that allows drainage of intramedullary blood to the knee joint after surgery if left open during surgery. Many studies claimed that sealing the intramedullary opening with a bone plug can decrease postoperative bleeding [5–8]. However, another study reported that sealing the intramedullary opening did not decrease blood loss or blood transfusion [9].

Due to the variety of results of available evidence, the authors aim to evaluate the effects of intramedullary sealing with a bone plug compared with leaving the opening open by a meta-analysis. The primary aim was to evaluate the transfusion rate, level of hemoglobin reduction, and drain volume.

## Methods

### Database search and search strategy

This systematic review and meta-analysis was performed following the PRISMA (preferred reporting items for systematic reviews and meta-analyses) Statement [10]. The following keywords and search combinations were used: (1) bone plug AND total knee arthroplasty (2) intramedullary sealing AND total knee arthroplasty. PubMed, Embase, Ovid, and the Cochrane Library were used for the search. Articles in any language published until May 2018 were included and analyzed.

### Study selection and data extraction

After the initial search, all article titles and abstracts were screened independently by two authors. The full manuscripts of relevant articles were evaluated for suitability by the inclusion criteria. The inclusion criteria were as follows: (1) the study compared the efficacy of intramedullary bone plug in TKA with an unplugged opening, (2) the study should be performed in primary TKA, (3) the study should evaluate postoperative bleeding aspects, and (4) the study was randomized and controlled. The studies that did not meet these criteria were excluded. In case of disagreements of both reviewers, a discussion between both reviewers was done to get a consensus. The intervention group included patients who had TKA with an intramedullary bone plug. The control group included patients who had TKA without an intramedullary bone plug. The primary outcome was blood transfusion rate. The secondary outcomes were drained volume of blood and the reduction in hemoglobin level.

### Risk of bias and quality assessment

Both reviewers independently assessed the risk of bias and the quality of the studies using the Cochrane risk-of-bias tool [11] to evaluate randomized control trials (RCTs). The *Cochrane Handbook for Systematic Reviews of Interventions* was used to evaluate the quality of the studies. The assessments were evaluated for random sequence generation, allocation concealment, blinding of participants and personnel, blinding of outcome assessment, incomplete outcome data addressed, selective reporting, and other biases.

### Statistical analysis

Review Manager (RevMan, version 5.3. Copenhagen: The Nordic Cochrane Centre, The Cochrane Collaboration, 2014) was used to conduct this meta-analysis. The transfusion rate was calculated with risk difference and 95% confidence interval. The drain volume and hemoglobin reduction were evaluated with standardized mean differences and 95% confidence interval. Heterogeneity was evaluated by *I*^2^. If *I*^2^ was greater than 50%, the data were considered to be statistically significant heterogeneity. The random-effects model was then used for data analysis. On the other hand, if *I*^2^ was less than 50%, the data analysis was done with the fixed-effects model.

### Search results

**Fig. 1:**
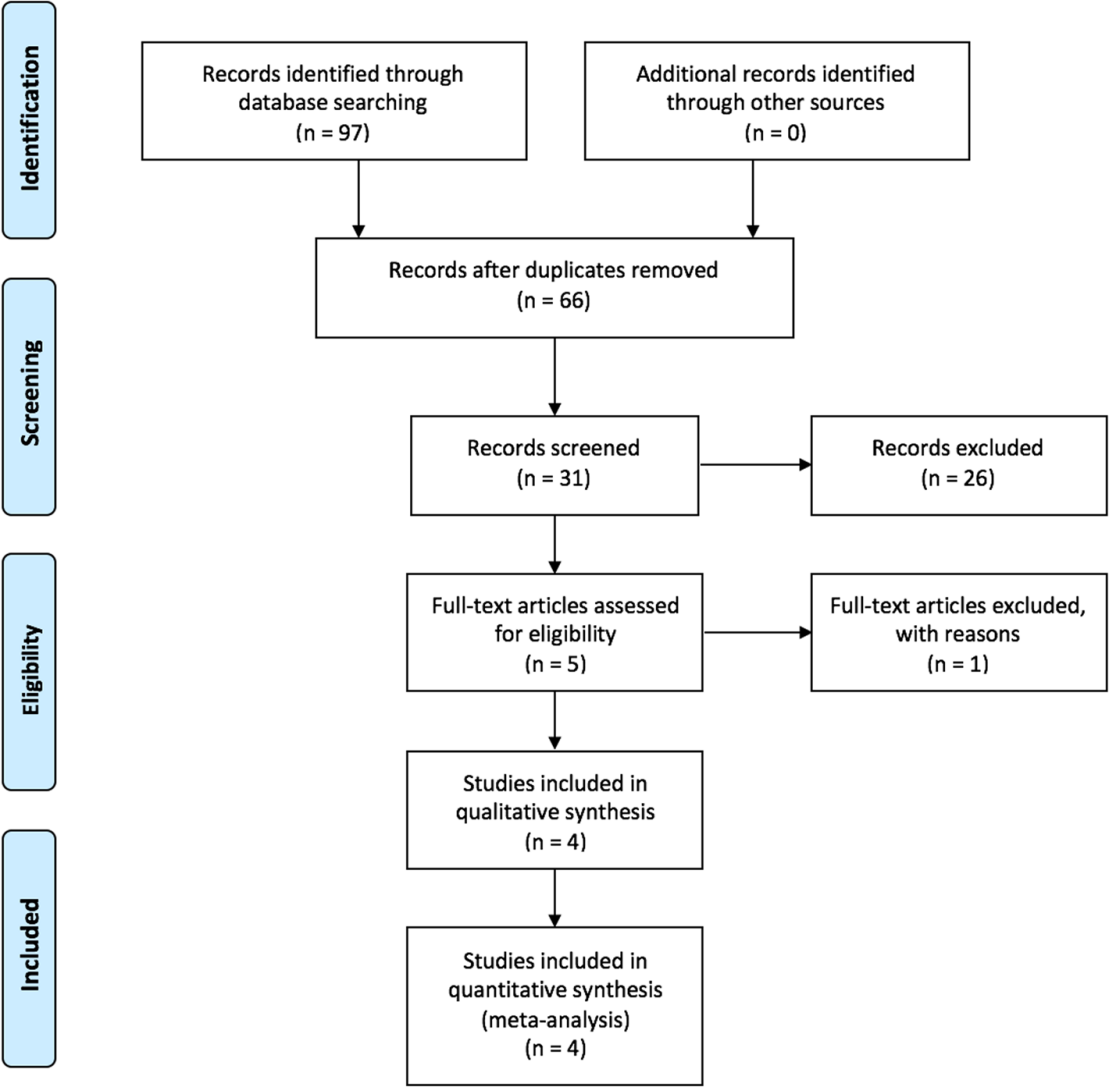
Flowchart of study selection

A total of 97 relevant studies were identified through the initial search. Thirty-one studies were excluded because they were duplicates. The remaining 66 studies were evaluated according to the inclusion criteria. Finally, four studies were included for a systematic review and meta-analysis. The flow chart diagram of the search for studies and selection is shown in Fig. 1. There were 320 patients in the intramedullary bone plug group and 316 patients in the “no plug” group.

### Study characteristics

The characteristics of the included studies are reported in Table 1. There were four RCTs. The total number of subjects from all studies was 636 patients. In all studies, one group of patients had an intramedullary bone plug while in the other group the intramedullary canal opening was left open.

**Table 1:** Characteristics of the included studies

Author	Year	Study type	Size	Cases		Age		Sex (F/M)		Type of prosthesis
				Plug	No plug	Plug	No plug	Plug	No plug	
Kumar et al. [7]	2000	RCTs	120	65	55	NA	NA	NA	NA	Cemented
Ko et al. [12]	2003	RCTs	262	128	134	NA	NA	98/30	100/34	Cemented
Torres-Claramunt et al. [6]	2014	RCTs	134	67	67	72.4	71.8	49/18	47/20	Cemented
Li et al. [5]	2017	RCTs	120	60	60	66.1	65.4	46/14	45/15	Cemented

### Risk of bias within studies

**Fig. 2:**
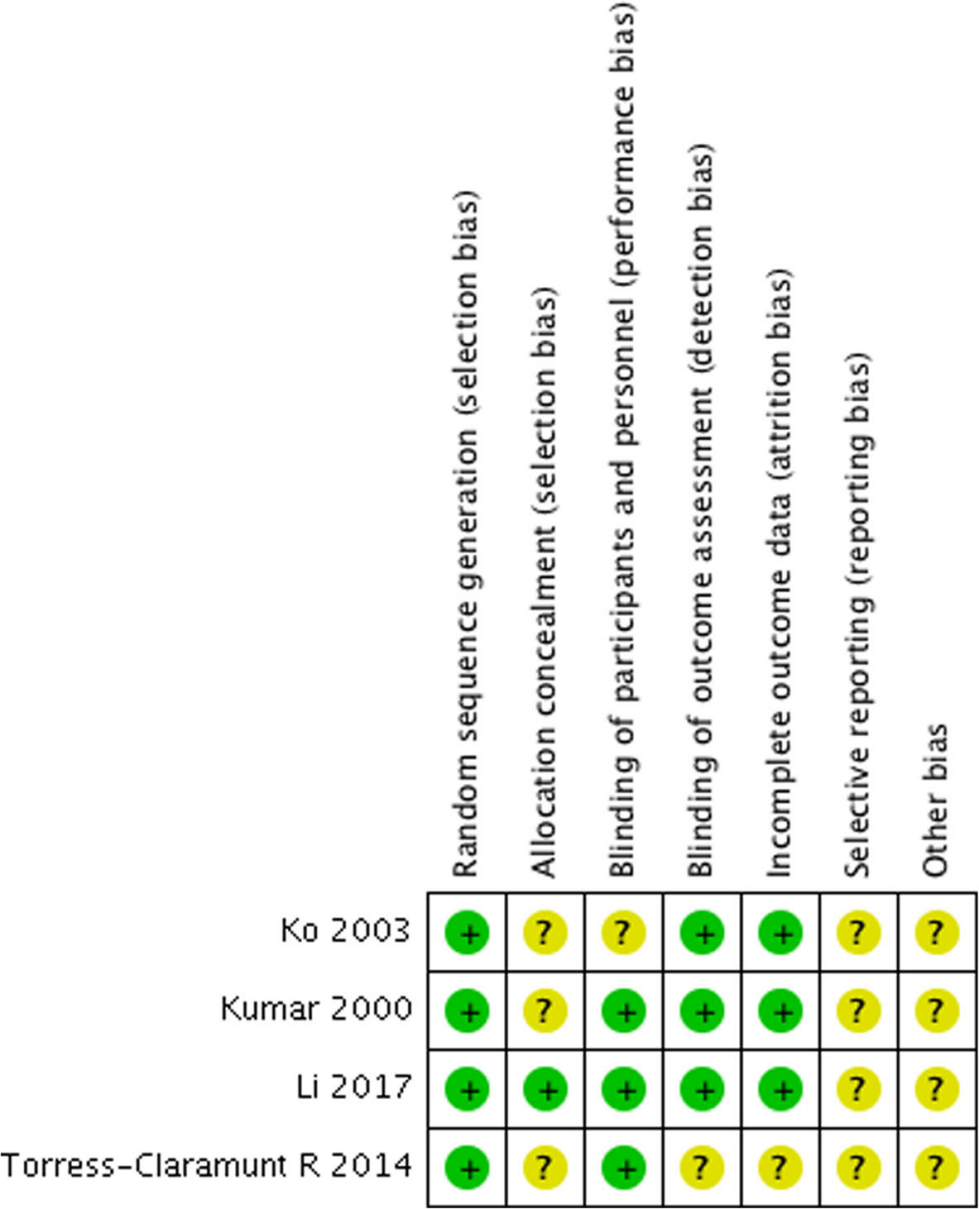
Risk of bias assessment of the included RCT studies

There were four RCTs. All studies used randomization methods. However, one study reported concealment of allocation [5]. Three studies reported blinding of the participants [5–7], and three studies blinded the assessors [5, 7, 12]. There were no incomplete outcome data results in any of the studies. Quality assessments of the studies were evaluated and are summarized in Fig. 2.

## Outcomes of the meta-analysis

### Transfusion rate

**Fig. 3:**
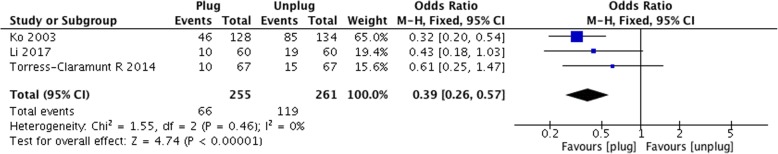
Forest plot for transfusion rate

Three studies with a total of 516 patients were evaluated for the postoperative transfusion rate comparisons between the intramedullary plug and no plug groups [5, 6, 12]. A fixed-effects model was used because homogeneity was found in the studies (*χ*^2^ = 1.55, [*p* = 0.46]; *I*^2^ = 0%). The pooled results demonstrated that patients with intramedullary plug had a lower rate of blood transfusion than patients with an unplugged intramedullary canal (*p* < 0.01) (Fig. 3).

### Hemoglobin reduction

**Fig. 4:**
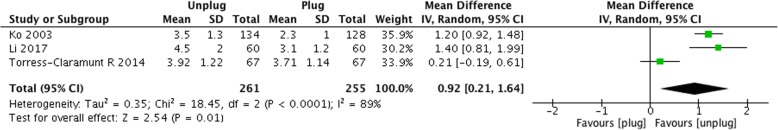
Forest plot for hemoglobin reduction

Hemoglobin reduction after TKA was reported in three studies with 516 patients [5, 6, 12]. There was significant heterogeneity in the pooled data (*τ*^2^ = 0.35; *χ*^2^ = 18.45, df = 2 [*p* < 0.01]; *I*^2^ = 89%). Therefore, a random-effects model was used to analyze the data. The analysis found that patients who had an open intramedullary canal had a higher level of postoperative hemoglobin reduction compared with patients in whom an intramedullary bone plug was used (*p* = 0.01) (Fig. 4).

### Drain volume

**Fig. 5:**
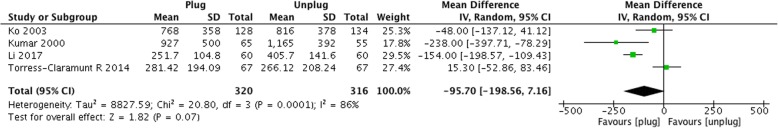
Forest plot of drain volume

Four studies with 636 patients reported the outcome of drain volume [5–7, 12]. There was statistical evidence of heterogeneity between the four studies (*τ*^2^ = 8827.59; *χ*^2^ = 20.80, [*p* < 0.01]; *I*^2^ = 86%). The patients who had intramedullary bone plug had no significant difference of drain volume comparing with patient who had an unplugged intramedullary canal (*p* = 0.07) (Fig. 5).

## Discussion

Postoperative blood loss after total knee replacement is a concern of surgeons. There are many methods to decrease postoperative blood loss. However, no study has reported which procedure was the most important step to reduce blood loss in TKA. Intramedullary bone plug is one of the options that claims to solve this problem by preventing bleeding from the intramedullary canal. Plugging the femoral intramedullary alignment hole with a bone graft is a simple procedure, and the bone graft is usually taken from the patient’s bone collected from the standard TKA bone cut procedure. However, the results varied in previous studies. Therefore, the authors aimed to perform a meta-analysis to evaluate postoperative blood loss from the effect of an intramedullary bone plug.

Blood transfusion is a concern after TKA. It was reported that patients who had postoperative blood transfusion had higher rates of surgical site infection and mortality [13–15]. The author’s meta-analysis demonstrated that using intramedullary bone plugs can reduce blood transfusion rates after TKA. Three studies compared the blood transfusion rates in plug and no plug groups. Most of the data demonstrated a lower incidence of transfusion in patients with an intramedullary bone plug compared with the no plug group. However, only two studies showed statistically significant differences [5, 12]. Postoperative complications were clearly reported in only two studies. Li et al. reported superficial infection in 2 of 60 patients in the intramedullary plug group and 3 of 60 patients in the no plug group [5]. Torres-Claramunt et al. also found superficial infection in two patients (one patient in each of two groups of 67 patients) and one deep infection in the no plug group [6].

The hemoglobin level reduction was lower in the intramedullary bone plug group in this meta-analysis. Two studies showed that the intramedullary bone plug group had a statistically significant lower reduction of the hemoglobin level [5, 12]. However, another study did not find a statistically significant difference between the two groups [6]. Most of the included studies found a lower mean postoperative drain volume [5, 7, 12]. However, the studies by Torres-Claramunt et al. [6] found a lower mean drain volume in the no plug group but there was no statistical difference. Our meta-analysis found that the intramedullary plug could not lower postoperative drain volume in TKA.

The strength of this meta-analysis is that it is the first meta-analysis to evaluate the effects of an intramedullary bone plug in primary TKA. However, the limitation of this study is that only four RCTs were included. The final outcome was possibly affected by the small sample size and some study bias. The second limitation was the large heterogeneity among the included studies.

## Conclusions

Intramedullary bone grafting into the femoral intramedullary guide opening may reduce the blood transfusion rate and hemoglobin level reduction. However, this meta-analysis had a limited number of RCT studies. Further high-quality large RCTs could provide more accurate comparison of this technique.

## Abbreviations

RCTs: Randomized control trials
TKA: Total knee arthroplasty
